# Domestic Ventilation

**Published:** 1901-11-02

**Authors:** 


### DOMESTIC VENTILATION.

The annual meeting of the Society of Medical Officers of Health was held on October 25th at the Hotel Cecil. The report of the council showed that forms for tabulating death returns had now been prepared on the lines laid down by the international commission, and it was hoped that their use would secure a universal system of classification comparable with that in use on the continent and in Canada and the United States. The president (Professor Wynter Blyth), speaking on the subject of ventilation, said that it had come as a surprise to many that not a drug in the pharmacopœia, not an animal extract, not one physical appliance, neither singly nor collectively, was equal in the treatment of tubercle to bathing the lungs and skin in fresh air. The remarkably successful treatment of consumptive maladies by fresh air must suggest to them that if air was curative it was also a preventative, and that if abundance of fresh air could be supplied to each unit of the population the death roll from phthisis would be so insignificant as to render special sanatoria unnecessary. The problem to be solved, he said, was not the changing of air three times an hour in the spacious rooms of the well-to-do, but in the small apartments sanctioned by the legislature —spaces of 400 or 300, or even of 250 cubic feet—for, obviously, if the problem was solved for the smaller it was also solved for the larger space. He further pointed out the distinct advance which had been made of late in the practice of ventilation by the wide distribution of electric energy, permitting such mechanical appliances as rotating fans to be utilised by ordinary householders. Wherever there was an electric supply, mechanical ventilation was cheap and practicable. Referring to the ventilation of railway tunnels, he said that the tubes were better than the underground, the air in them being, in fact, better than that in ordinary places of assembly—a statement which, we may add, does not say much.

